# Fangchinoline attenuates hepatic fibrosis by regulating taurine metabolism and oxidative stress

**DOI:** 10.3389/fphar.2025.1633519

**Published:** 2025-08-20

**Authors:** Hui Yin, Hang Lian, Yawen Wang, Luoting Chen, Xueting Liu, Yange Liu

**Affiliations:** ^1^ School of Basic Medical Sciences, Jiangxi Medical College, Nanchang University, Nanchang, Jiangxi, China; ^2^ Department of Thoracic Surgery, The First Affiliated Hospital of Shaoyang University, Shaoyang, Hunan, China

**Keywords:** fangchinoline, hepatic fibrosis, taurine, oxidative stress, hepatic stellate cells

## Abstract

**Background:**

Hepatic fibrosis emerges as a pathological hallmark in the pathogenesis of chronic hepatopathies. *Stephania tetrandra S. Moore* is a traditional Chinese herb used to treat liver disease. However, the anti-hepatic fibrosis effect of fangchinoline (FAN), an active ingredient of *S. tetrandra S. Moore*, has not been reported. This study aimed to elucidate the anti-hepatic fibrosis effect of FAN and clarify the underlying molecular mechanisms.

**Methods:**

The DEN-induced hepatic fibrosis mouse model, primary murine hepatic stellate cells (HSCs), and TGF-β-induced activation model of HSCs were used to explore the anti-fibrotic effect of FAN. The proteomics analysis was used to predict the pharmacodynamic mechanisms of FAN, and follow-up validation assays were performed with *in vivo* and *in vitro* experiments.

**Results:**

FAN alleviated DEN-induced liver fibrosis in mice, reducing biomarker levels, slowing histopathological changes, and inhibiting collagen deposition. FAN suppressed HSCs activation and the biosynthetic abilities of the extracellular matrix. Proteomics was used to explore the mechanisms of FAN action, which is related to the regulation of taurine metabolism. FAN reversed DEN-induced changes in the levels of taurine and key enzymes that catalyze taurine synthesis. Additional taurine reinforces the regulatory effect of FAN on HSCs activation. Taurine could inhibit oxidative stress. FAN reduced DEN-induced ROS accumulation, which may be associated with Nrf2 pathway activation. Cleaning ROS with N-acetylcysteine enhanced the anti-fibrotic effects of FAN.

**Conclusion:**

FAN can alleviate hepatic fibrosis by regulating taurine metabolism and oxidative stress, which has an important theoretical value.

## Highlights

- The antifibrotic effect of FAN was first confirmed in mouse and cell models.
- Taurine metabolism and oxidative stress are exploited as the potential targets for hepatic fibrosis therapy.
- This paper found that regulating taurine metabolism and oxidative stress are the likely mechanisms underlying the protective effects of FAN against hepatic fibrosis.

## 1 Introduction

Hepatic fibrosis, a severe global health problem, is an evolving, multifaceted tissue process response following chronic liver damage caused by several stimuli including viruses, alcohol, metabolic disorders, autoimmune diseases, chronic biliary stasis, or hypoxia (Lotersztajn et al., 2005). Progressive hepatic fibrosis may escalate to cirrhosis, ultimately predisposing patients to risk of hepatocellular carcinoma (HCC) or end-stage liver disease.

Chronic liver injury disrupts tissue repair by inducing pathological extracellular matrix (ECM) remodeling, where the overproduction of key components (collagens, proteoglycans, and glycoproteins) overwhelms their degradation capacity, perpetuating fibrotic progression (Parola and Pinzani, 2019). Type-I and -III fibrillar collagens accumulate in the liver, leading to the formation and thickening of fibrotic membranes, cross-linking of collagen, and structural distortion of the liver. Myofibroblasts (MFs) are an important source of extracellular matrix synthesis and secretion, and they also express abundant intracellular proteins such as vimentin, non-muscle actin, and α-smooth muscle actin (α-SMA) (Schuster et al., 2023). Myofibroblasts are mainly transformed from activated hepatic stellate cells (HSCs) (George et al., 2019).

Current anti-fibrotic approaches focusing on suppressing of the scarring response mainly include (1) inhibition of HSCs activation; (2) reduction of fibroblast accumulation through growth-inhibiting or pro-apoptotic compounds; (3) reducing extracellular matrix synthesis or enhancing its degradation. Injured and apoptotic hepatocytes drive hepatic stellate cell activation via multiple mechanisms, including immune cell infiltration, reactive oxygen species (ROS) generation, and secretion of fibrosis-promoting factors (Hao et al., 2024).

Therefore, HSCs activation during chronic liver disease is potentially driven by redox imbalance within the fibrotic microenvironment. Oxidative stress arises from disrupted redox homeostasis, where excessive ROS generation overwhelms the endogenous antioxidant defenses, thus driving pathological cellular damage. ROS comprise oxygen-derived reactive molecules, including superoxide anion, hydrogen peroxide, hydroxyl radical, and nitric oxide (Hayes et al., 2020). Both liver-specific resident macrophages (Kupffer cells) and liver-infiltrating neutrophils can stimulate HSCs activation through ROS production (Luangmonkong et al., 2018). Oxidative stress inhibition by the aldo-keto reductase family 7 member A2 underpins glycyrrhizic acid’s therapeutic action against liver fibrosis (Wang Q. et al., 2024). Curcumin combats hepatic fibrosis *via* epithelial–mesenchymal transition suppression in hepatocytes, which is mediated by oxidative stress and autophagy (Kong et al., 2020).

*Stephania tetrandra S. Moore* is utilized in traditional herbal medicine across China, Japan, and South Korea, where it is standardized as “Fang ji” in the China Pharmacopoeia Commission (2015). *Stephania tetrandra S. Moore*, colloquially referred to in Chinese folk medicine as “Fen Fang ji,” “Han Fangji,” or “Guang Fangji,” was first documented as a medicinal herb in the “*Shen Nong Ben Cao Jing.*” With a history of therapeutic use spanning over a millennium, classical Chinese medical texts such as the “*Ben Cao Gang Mu*” and “*Ben Cao Cong Xin*” describe its applications for treating wind-cold, wind-swelling, rheumatoid arthritis, and inflammatory sores, underscoring its anti-inflammatory properties (Jiang et al., 2020). The release of inflammatory factors triggered by hepatocyte injury induced through diverse etiologies (e.g., viral infections, alcohol abuse, and metabolic dysregulation) serves as a key driver in HSCs activation and collagen deposition during hepatic fibrogenesis (Zhang and Friedman, 2012). Therefore, *S. tetrandra S. Moore* may hold therapeutic potential for hepatic fibrosis treatment owing to its potent anti-inflammatory properties. Evidence suggests that *S. tetrandra S. Moore* exhibits anti-fibrotic effects in hepatic fibrosis (Chor et al., 2009). Fangchinoline (FAN), isolated from *S. tetrandra S. Moore* roots, is a bis-benzylisoquinoline alkaloid with antitumor properties (Liu et al., 2024), anti-viral effect (Wang J. et al., 2024), and anti-arthritic effect (Villa et al., 2020). Therefore, FAN may hold significant therapeutic potential for hepatic fibrosis treatment; however, no detailed mechanistic studies supporting this application have been reported to date.

This study investigates the anti-fibrotic effects of FAN and delineates the mechanistic basis.

## 2 Materials and methods

### 2.1 Antibodies and reagents

Reagents: Fangchinoline (≥98%, B50673) was ordered from Shanghai Yuanye Bio-Technology (Shanghai, China). N-Acetylcysteine (NAC, HY-B0215) and diethylnitrosamine (DEN, HY-N7434) were sourced from MCE (New Jersey, USA). Commercial ELISA kits for aspartate aminotransferase (AST), alanine aminotransferase (ALT), and alkaline phosphatase (ALP) were sourced from Jiangsu Kete Biotechnology (Jiangsu, China). The Nanjing Jiancheng Bioengineering Institute (Nanjing, China) provided the ROS assay kit. ELISA kits for taurine were sourced from Zhonghao Biotechnology (Beijing, China).

Antibodies: The rabbit anti-nuclear factor erythroid 2-related factor 2 (Nrf2, 16396-1-AP), anti-superoxide dismutase 1 (SOD1, 10269-1-AP), anti-β-actin (66009-1-lg) antibodies, anti-glyceraldehyde-3-phosphate dehydrogenase (GAPDH, 10494-1-AP) antibodies, anti-catalase (CAT, 21260-1-AP) and the mouse anti-kelch-like ECH-associated protein 1 (Keap1, 60027-1-Ig), and anti-heme oxygenase-1 (HO-1, 66743-1-Ig) antibodies were sourced from Proteintech (Wuhan, China). The rabbit anti-collagen type I alpha 1 (COL1A1 and A25439), anti-collagen type I alpha 2 (COL1A2 and A5786), anti-α-SMA (66009-1-lg), and anti-cysteine sulfinic acid decarboxylase (CSAD and A13845) antibodies were ordered from ABclonal (Wuhan, China). The rabbit anti-collagen III alpha 1 antibodies (COL3A1 and sR23957) and anti-α-SMA (R380653) were ordered from Zen-Bio (Chengdu, China).

### 2.2 DEN-induced mouse model of hepatic fibrosis

The animal studies strictly complied with the guidelines of the Institutional Animal Ethics Committee of Nanchang University (approval No. SYXK(Gan)2021-0004).

#### 2.2.1 Design 1

Male C57BL/6 mice (3-week-old) were provided by Changsheng Biotechnology (Liaoning, China). After 7 days of acclimatization, the mice were randomly divided into two groups. In addition to the vehicle control group (n = 6), the remaining mice were intraperitoneally injected with DEN at 10 mg/kg for the first week, 25 mg/kg DEN for the second week, and 30 mg/kg DEN every week from the third week to the 18th week, to induce hepatic fibrosis. Vehicle control mice (n = 6) were intraperitoneally injected with saline. From the fourth week, mice injected with DEN were randomly divided into three groups (n = 6 per group): 10 mL/kg saline, 10 mg/kg FAN, or 20 mg/kg FAN were administered to mice every 2 days, respectively. Every 3 weeks, the body weight was tracked to monitor changes. In the 18th week, the experiment was completed, and the serum and tissues (the liver, spleen, lung, heart, kidney, and thymus) were collected.

$$\text{The organ index was expressed as}:\text{Organ index}=\frac{\text{organ weight}}{\text{body weight}\;}\times 100\%.$$ (1)

#### 2.2.2 Design 2

Male C57BL/6 mice (3-week-old) were provided by Changsheng Biotechnology (Liaoning, China). DEN was used to create a mouse model of liver fibrosis according to the protocol of “Design 1.” From the fourth week onward, all mice were randomly allocated into five groups (n = 6 per group) and received intraperitoneal injections every 2 days as follows: physiological saline (DEN control group), 10 mg/kg FAN alone, 10 mg/kg FAN plus 100 mg/kg NAC (alternating administration), 20 mg/kg FAN alone, and 20 mg/kg FAN plus 100 mg/kg NAC (alternating administration). The treatment regimen was maintained throughout the 18-week period, with body weight monitoring and terminal euthanasia performed upon experimental completion. Serum and tissue samples (the liver, spleen, lung, heart, kidney, and thymus) were harvested. Following organ weighing, the organ index was determined according to Equation 1.

### 2.3 Histopathological examination

The fixed liver, kidney, and lung tissues underwent sequential processing: dehydration through graded ethanol–xylene solutions, paraffin embedding, and microtome sectioning. Tissue sections were subsequently stained with hematoxylin and eosin (H&E), and histological imaging was performed using a microscope.

### 2.4 Picrosirius red staining and Masson’s trichrome staining

Liver sections underwent standard histological processing (deparaffinization and rehydration) prior to Picrosirius red staining. The slices were stained with Picrosirius red, dehydrated using alcohol, and subsequently visualized using a microscope.

For Masson’s trichrome staining, paraffin-processed hepatic tissue was cut into 5-μm slices and conventionally deparaffinized. The sections were incubated with potassium bichromate overnight and stained successively with hematoxylin, Ponceau S acid fuchsin stain, phosphomolybdic acid, and aniline blue. Slides were dehydrated and coverslipped under Permount mounting medium. Photographs were captured with a microscope (Olympus, Tokyo, Japan).

### 2.5 Biochemical assays

Liver tissues from the designated groups were homogenized, followed by centrifugation. The supernatants and serum were tested for ROS, ALT, AST, ALP, and taurine levels using commercial assay kits as per the manufacturers' protocols.

### 2.6 Ultra-fast quantitative proteomics analysis

Liver samples from mice in the blank control and model control groups (three livers per group) were ground, lysed, and centrifuged, and the supernatant was collected. Protein quantification with the BCA assay preceded sample normalization for equal loading. The protein solution was hydrolyzed by protease and desalted by using a C18 column. Samples were separated using a Vanquish Neo UHPLC nanoflow system (Thermo Fisher Scientific). The mobile phases consisted of phase A: 0.1% (v/v) formic acid in water and phase B: 0.1% (v/v) formic acid in acetonitrile (100% acetonitrile). Separation was carried out using a capture-analytical dual-column configuration: trap column: PepMap Neo Trap Cartridge (300 μm × 5 mm, 5 μm); analytical column: Easy-Spray™ PepMap™ Neo UHPLC column (150 μm × 15 cm, 2 μm). The analytical column was maintained at 55 °C. Samples (200 ng) were loaded at a flow rate of 2.5 μL/min. Chromatographically separated samples were analyzed *via* data-independent acquisition (DIA) using an Orbitrap Astral mass spectrometer (Thermo Fisher Scientific) coupled to the nanoflow Vanquish Neo system. MS parameters included the following: ion mode: positive; MS1 scan range: 380 m/z–980 m/z; MS1 resolution: 240,000 at 200 m/z; normalized AGC target: 500%; maximum injection time: 5 m. The DIA settings included the following: MS2 scans: 299 variable windows; isolation window: 2 Th; HCD collision energy: 25%; normalized AGC target: 500%; maximum injection time: 3 ms.

The proteins in each group were quantified, and the results of the statistical analysis were used to screen out the differentially expressed protein. The principal component analysis (PCA) was performed on the samples to assess both the overall protein differences among sample groups and the magnitude of variation within each group. Proteins with either fold change (FC) ≥ 1.5 or FC ≤ 0.6667 and p-value ≤0.05 were defined as differentially expressed proteins in the two-sample comparison groups, where FC was calculated as the ratio of the average expression in the DEN-treated group to that in the control group. Functional annotation and volcano plot of the identified differential proteins were performed.

### 2.7 Cell culture

Primary activated murine HSCs (MIC-iCell-d018) were sourced from iCell Bioscience (Shanghai, China) and maintained in DMEM (Solarbio, Beijing, China) containing 10% FBS (ExCell Bio, Shanghai, China) and penicillin/streptomycin, and it was maintained under standardized conditions (37 °C, 5% CO_2_, and humidified atmosphere).

Human hepatic stellate cells (LX-2 cells, SCSP-527) were purchased from the Cell Bank of Chinese Academy of Sciences Committee (Shanghai, China), cultured in a specialized medium (SNLM-206, SUNNCELL, Wuhan, China), and maintained under standardized conditions.

### 2.8 Immunofluorescence analysis

LX-2 cells were treated with 20 ng/mL TGF-β for 24 h or 48 h, followed by incubation with 12.5 μM FAN with or without 8 mM NAC for 24 h. After fixation with 4% paraformaldehyde and permeabilization, cells were incubated overnight at 4 °C with primary antibodies against COL1A2 or α-SMA. Following thorough washing, fluorophore-conjugated secondary antibodies were applied. The nuclei were counterstained with DAPI, and the fluorescence signal distribution was visualized under a fluorescence microscope.

### 2.9 Cell morphology analysis

For the analysis of cell morphology, the primary murine HSCs were exposed to 6.25 μM or 12.5 μM FAN with or without 5 mM taurine for 24 h. From among those cells, the group treated with taurine alone was used as the positive control. Cell morphology was observed by light microscopy.

For phalloidin staining, adherent cells on glass substrates were exposed to 6.25 μM or 12.5 μM FAN with or without 5 mM taurine for 24 h. Following fixation with paraformaldehyde and permeabilization, samples were co-stained with phalloidin and DAPI and then examined using a Diaphot fluorescence microscope.

### 2.10 Western blot

Protein lysates from murine tissues and primary HSCs were resolved *via* 10%–15% gradient sodium dodecyl sulfate–polyacrylamide gel electrophoresis. After electrophoretic transfer onto membranes, they were blocked and incubated in primary antibodies including COL1A1, COL1A2, COL3A1, α-SMA, CSAD, Keap1, Nrf2, CAT, HO-1, SOD1, GAPDH, or β-actin, and then they were exposed to horseradish peroxidase-conjugated secondary antibodies. Protein band visualization was achieved using a chemiluminescence detection system coupled with the imaging platform. Quantitative analysis of band intensities was performed through pixel density measurements *via* ImageJ software.

### 2.11 Statistical analysis

Statistical analyses were performed using SPSS 22.0 and GraphPad Prism 8.0. Data were presented as the mean ± the standard deviation (SD). Multi-group comparisons were analyzed by one-way ANOVA (*p* < 0.05 was considered significant).

## 3 Results

### 3.1 FAN ameliorates hepatic dysfunction and fibrosis in the DEN-induced mice model

**FIGURE 1:**
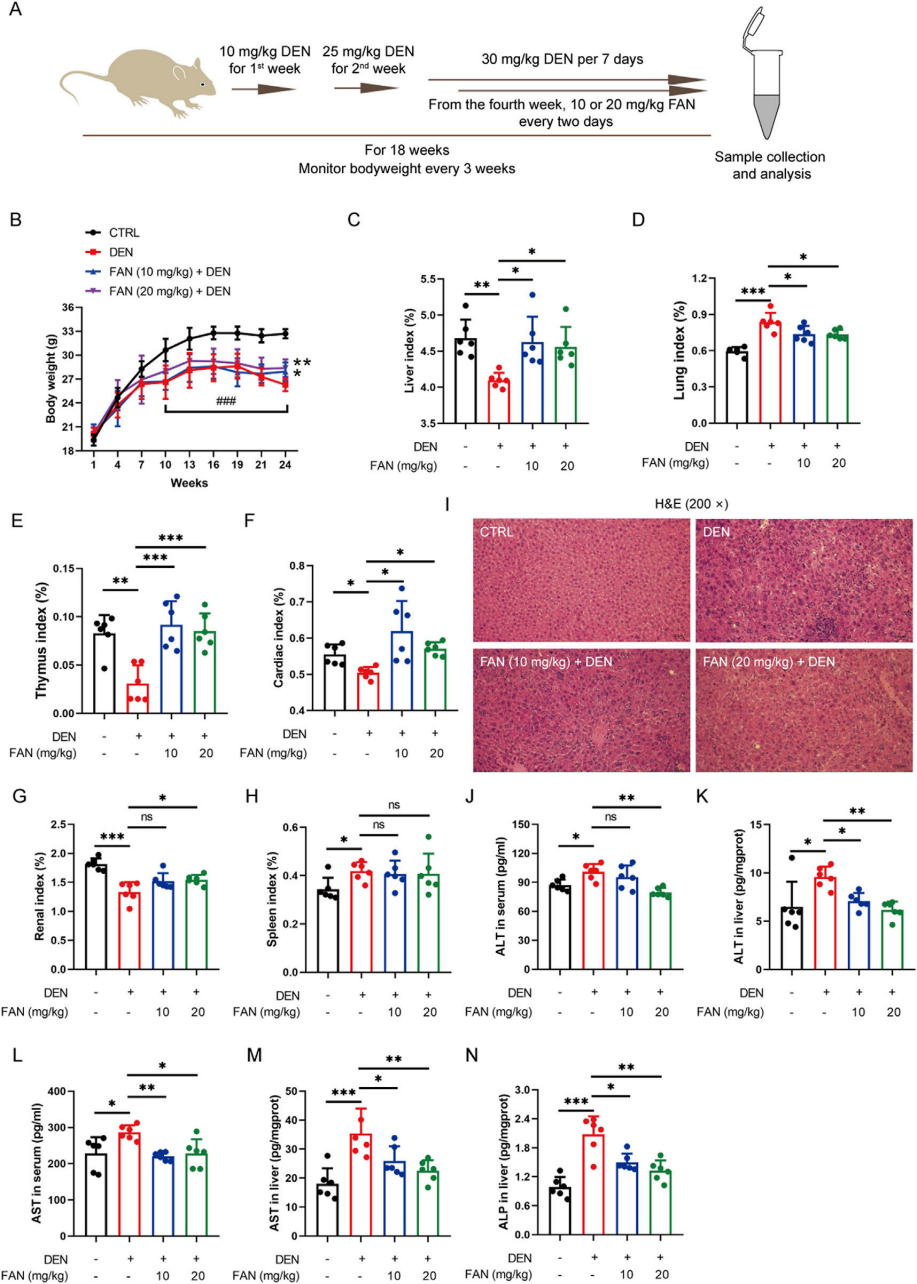
FAN depresses DEN-induced liver injury. **(A)** Diagram of the experimental plan for developing the DEN-induced hepatic fibrosis model and drug treatment (n = 6). **(B)** Body weight was monitored every 3 weeks. **(C–H)** Indices of the liver **(C)**, lung **(D)**, thymus **(E)**, heart **(F)**, kidney **(G)**, and spleen **(H)** were measured at the time of sacrifice. **(I)** Mouse liver was stained with H&E. Scale bar, 20 μm. **(J–N)** Serum or liver ALT, AST, and ALP levels were detected. Data are presented as the means ± S.D. ^*^ *P* < 0.05, ^**^ *p* < 0.01, and ^***^ *p* < 0.001; ns, not significant.

To investigate FAN’s hepatoprotective effects against fibrosis, we employed a DEN-induced mouse model replicating human hepatic fibrogenesis, with the experimental schematic detailed in Figure 1A. FAN significantly alleviated DEN-induced loss of bodyweight and liver index in mice (Figure 1B, C; Supplementary Figure S1A). In addition, FAN significantly alleviated DEN-induced changes of the lung index, thymus index, cardiac index, and renal index, but not the spleen index (Figures 1D–H). Hepatic biomarker panel abnormalities (ALT, AST, and ALP) can be regarded as a significant event of liver disease. DEN administration increased serum or liver ALT, AST, and ALP levels, but FAN could prevent these changes (Figures 1J–N).

**FIGURE 2:**
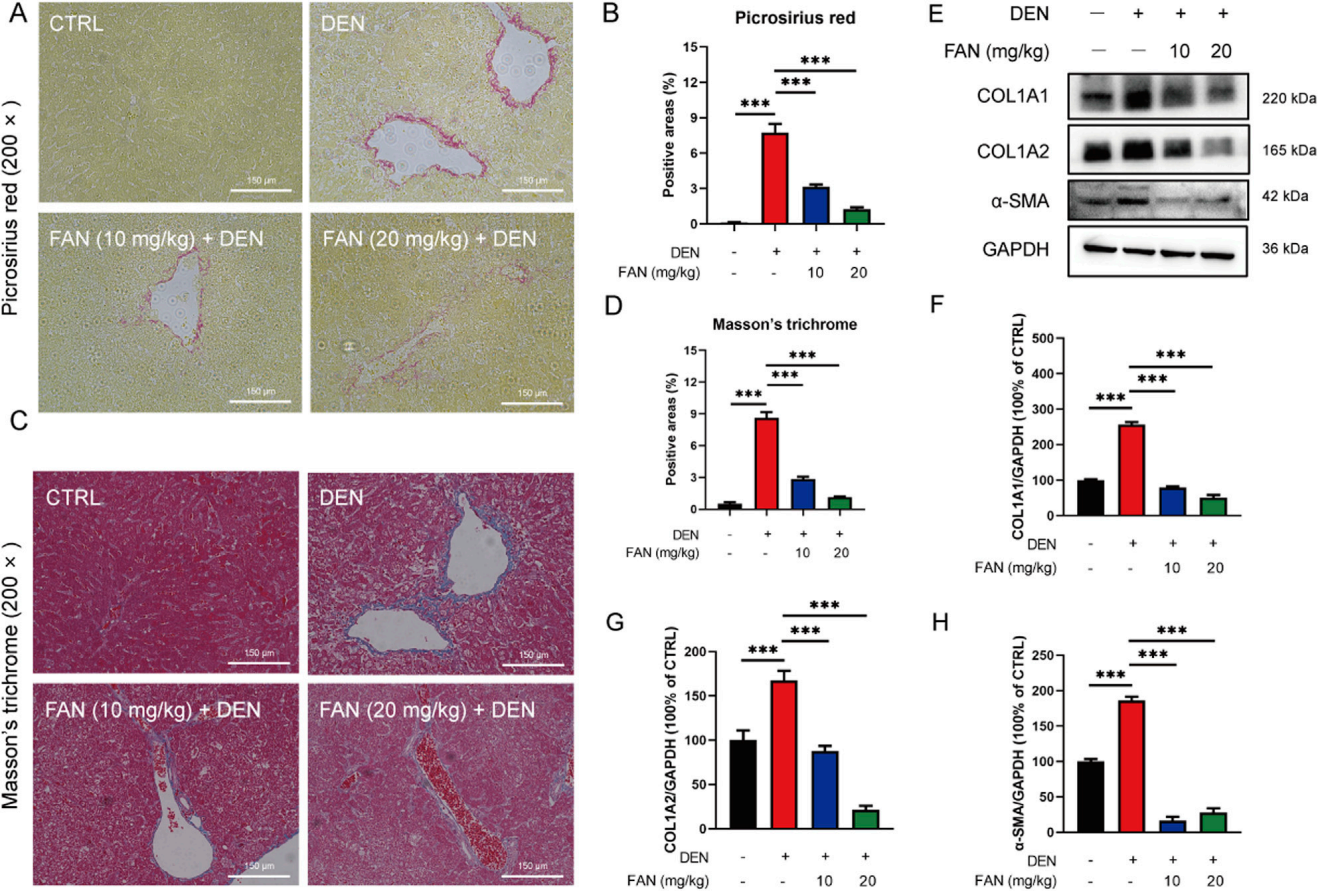
FAN inhibits DEN-induced hepatic fibrosis *in vivo*. **(A–D)** Liver tissues of mice were subjected to Picrosirius red staining **(A)** or Masson’s trichrome **(C)**, and quantitative analysis was performed on the positively stained areas **(B, D)**. Scale bar, 150 μm; n = 3. **(E–H)** Expressions of the indicated proteins in the liver were detected by Western blot **(E)**, and the relative expressions of the indicated proteins over GAPDH were quantified **(F–H)**; n = 3. Data are presented as the means ± S.D. ^***^ *P* < 0.001.

Histopathology confirmed that FAN treatment markedly mitigated the hydropic degeneration, swelling, and necrosis of liver cells; desmoplasia; and scattered inflammatory cell infiltration around many veins in the liver (Figure 1I); it also mitigated bronchial luminal narrowing in the lung (Supplementary Figure S1B) and mesangial expansion and diffuse thickening of the glomerular basement membrane (Supplementary Figure S1C). Picrosirius red is an acid dye that binds easily to the basic groups in collagen molecules and is used to detect the deposition of collagen molecules in tissues. Masson’s trichrome staining is also a classic method to visualize the structure of collagen in pathological tissues. At 18 weeks after DEN injection, Picrosirius red staining and Masson’s trichrome staining detected severe collagen accumulation in hepatic tissues of the DEN-treated only group. These changes exhibited significant attenuation following FAN administration (Figures 2A–D). Thus, FAN can alleviate DEN-induced hepatic fibrosis.

### 3.2 FAN decreases DEN-induced expression of matrix proteins in liver and HSCs activation

**FIGURE 3:**
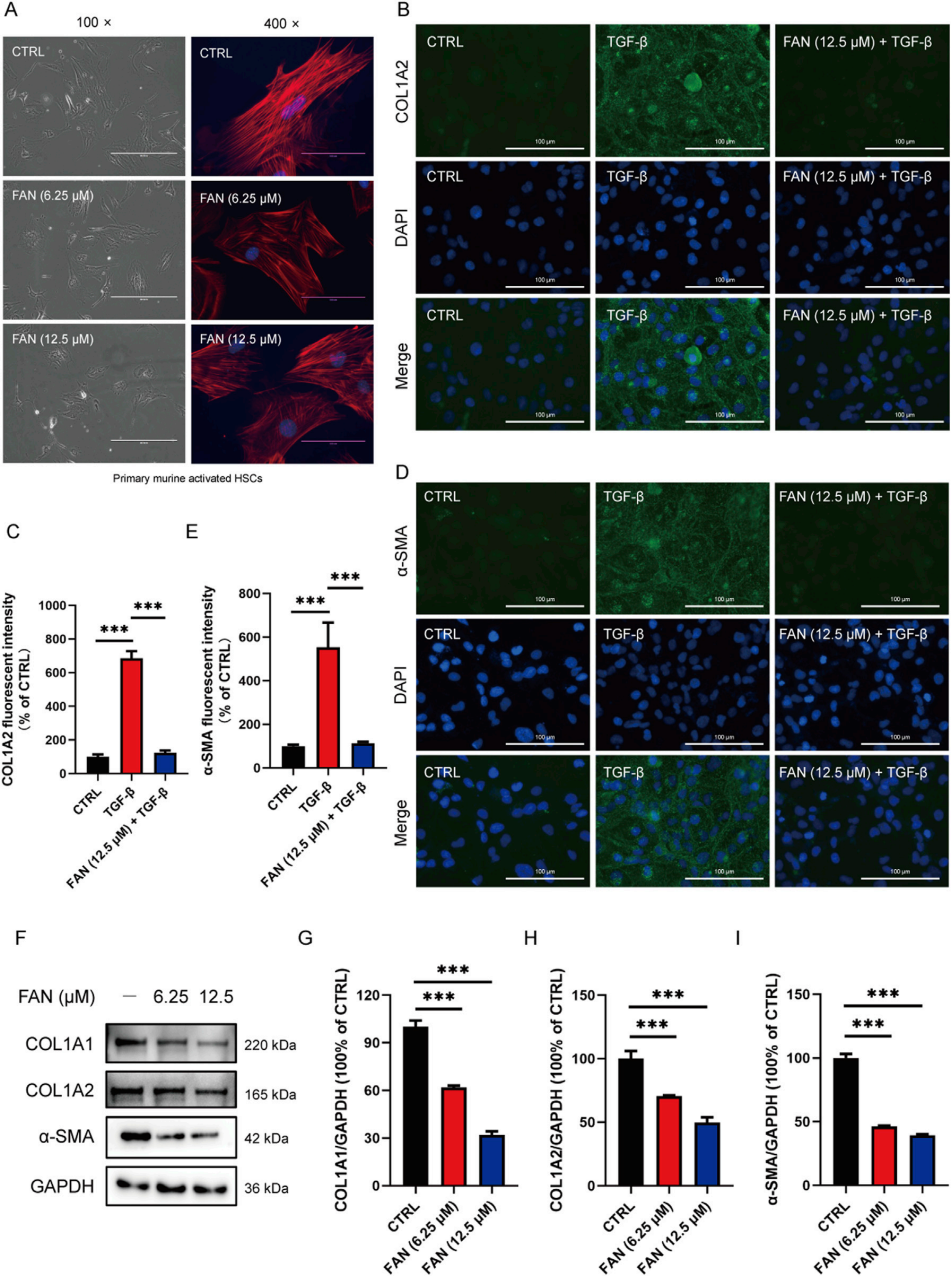
FAN inhibits DEN-induced hepatic fibrosis *in vitro*. **(A)** Primary murine activated HSCs were exposed to 6.25 μM or 12.5 μM FAN. After incubation for 24 h, some of the cells were photographed (left panel; scale bar, 400 μm), and the others were fixed, stained with phalloidin and DAPI, and photographed (scale bar, 100 μm). **(B–E)** LX-2 cells were treated with TGF-β (20 ng/mL) for 24 h to establish an activated HSCs model. Following treatment with or without 12.5 μM FAN for 24 h, the expressions of COL1A2 **(B)** and α-SMA **(D)** were detected by immunofluorescence and quantitatively analyzed **(C, E)**. Scale bar, 100 μm; n = 3. **(F–I)** Expressions of the indicated proteins in primary murine activated HSCs were detected by Western blot **(F)**, and the relative expressions of indicated proteins over GAPDH were quantified **(G–I)**; n = 3; ^***^ *p* < 0.001.

Excessive synthesis and accumulation of liver matrix proteins contribute to collagen deposition. DEN administration markedly upregulated hepatic profibrotic markers (COL1A1, COL1A2, and α-SMA) *versus* that in controls, an effect substantially attenuated by FAN intervention (Figures 2E–H). Activated HSCs can turn into myofibroblasts, which are the main source of extracellular matrix synthesis and secretion. We obtained activated HSCs from the mouse liver. Primary murine activated HSCs have the characteristics of myofibroblasts, showing that the cells tend to be spindle-shaped. Cells were either treated with FAN or left untreated, and their morphology was examined. After FAN administration, the morphology of activated HSCs lost the spindle-shaped appearance, which suggests that HSCs activation was inhibited (Figure 3A). We treated LX-2 cells with TGF-β to establish an activated model of HSCs. The effect of FAN treatment on the extracellular matrix protein levels was assessed using immunofluorescence. It was found that 12.5 μM FAN significantly suppressed TGF-β-induced increases in COL1A2 and α-SMA levels in LX-2 cells (Figures 3B–E). Western blot analysis similarly demonstrated that FAN markedly inhibited the high levels of COL1A1, COL1A2, and α-SMA in activated HSCs (Figures 3F–I). These results further demonstrate that FAN can relieve hepatic fibrosis *via* decreasing the matrix protein expression *in vivo* and *in vitro*.

### 3.3 FAN inhibits DEN-induced reduction in the synthesis of taurine

**FIGURE 4:**
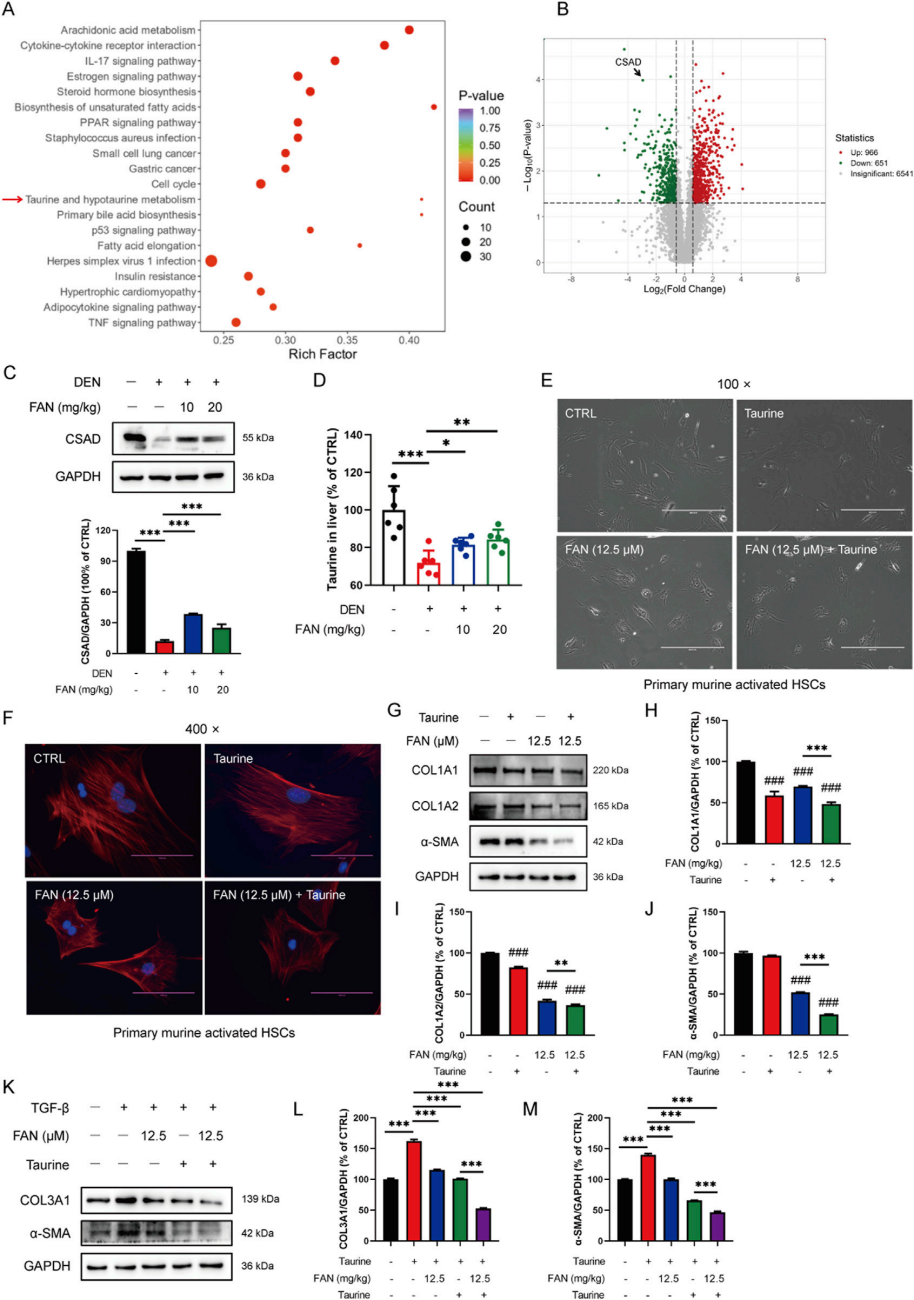
FAN mediates taurine metabolism, and the addition of taurine enhances the anti-fibrotic effect of FAN. **(A, B)** Differential proteins between the control group and model group were screened using ultra-fast quantitative proteomic techniques; n = 3. The top-20 GO-terms of *p*-value in the enrichment analysis were selected to draw the bubble diagram **(A)**; fold change of differential proteins was logarithmic at the base of 2, and the absolute value of *p*-value was logarithmic at the base of 10, and the volcano plot was drawn **(B)**. **(C)** Expression of CSAD in the liver was detected by Western blot (upper panel), and the relative expressions of indicated proteins over GAPDH were quantified (lower panel); n = 3. **(D)** Expression of taurine in the liver was detected by a kit; n = 6. **(E, F)** Primary murine-activated HSCs were exposed to FAN or/and taurine. The treated cells were photographed; scale bar, 400 μm; n = 3 **(E)**. Treated cells were stained with Picrosirius and DAPI and photographed; scale bar, 100 μm; n = 3 **(F)**. **(G–J)** Expressions of the indicated proteins in primary murine-activated HSCs were detected by Western blot **(G),** and the relative expressions of indicated proteins over GAPDH were quantified; n = 3 **(H–J)**. (K–M) LX-2 cells were treated with TGF-β (20 ng/mL) for 48 h. Following treatment with or without 12.5 μM FAN for 24 h, the expressions of COL3A1 and α-SMA were detected by Western blot **(K)**, and the relative expressions of indicated proteins over GAPDH were quantified; n = 3 **(L, M)**. Data are presented as the means ± S.D. ^###^ *P* < 0.001 in a comparison with the control group; ^**^ *p* < 0.01, ^***^ *p* < 0.001; ns, not significant.

To further clarify which molecular mechanism FAN’s anti-fibrotic effect depends on, we used ultra-fast quantitative proteomic techniques to screen for differential proteins between the control and model groups. KOG analysis revealed that DEN could significantly affect taurine and hypotaurine metabolism, unsaturated fat synthesis, and arachidonic acid metabolism (Figure 4A). Using the volcano plot to quickly identify the significantly different proteins in two groups, we found that the enzyme CSAD, a key enzyme in taurine synthesis, was significantly reduced (Figure 4B). The hepatic CSAD level was decreased by DEN, and this value was increased by treatment with FAN (Figure 4C). Taurine levels in the mouse liver were measured using a kit. It was found that the taurine levels were significantly reduced in mice with hepatic fibrosis compared to the control group. FAN treatment effectively reversed this reduction (Figure 4D).

### 3.4 Additional taurine reinforces the anti-fibrotic effect of FAN

To investigate whether FAN could also inhibit hepatic fibrosis by modulating the synthesis of taurine, we exposed activated primary murine HSCs with or without taurine and examined the morphological modifications of cells and the matrix protein expression levels in cells. Clinical trials demonstrated taurine’s therapeutic potential in chronic liver disease, especially in painful muscle spasms in patients with liver disease (Vidot et al., 2018). The activated primary murine HSCs acquired myofibroblast-like features. On the one hand, we chose taurine as a tool to investigate the pharmacological mechanism of FAN, and on the other hand, we chose taurine as a positive control agent (H et al., 2018; Tapper and Parikh, 2023). The activation of HSCs was inhibited by treatment with FAN plus taurine compared with the FAN monotherapy group (Figures 4E, F). We found that the elevated expressions of COL1A1, COL1A2, and α-SMA in activated HSCs were decreased after FAN treatment. Additional taurine further reduced the expressions of the three proteins following FAN treatment (Figures 4G–J). Similarly, FAN effectively attenuated the TGF-β-induced increases in COL3A1 and α-SMA protein levels in LX-2 cells. Furthermore, compared to FAN treatment alone, the combination treatment with taurine and FAN further enhanced the ability of FAN to reduce the extracellular matrix protein levels in activated LX-2 cells (Figures 4K–M). Therefore, we reveal that FAN can inhibit hepatic fibrosis by increasing taurine content by raising the protein levels of CSAD, a key enzyme of taurine synthesis.

### 3.5 Inhibition of oxidative stress enhances the anti-fibrotic effects of FAN

**FIGURE 5:**
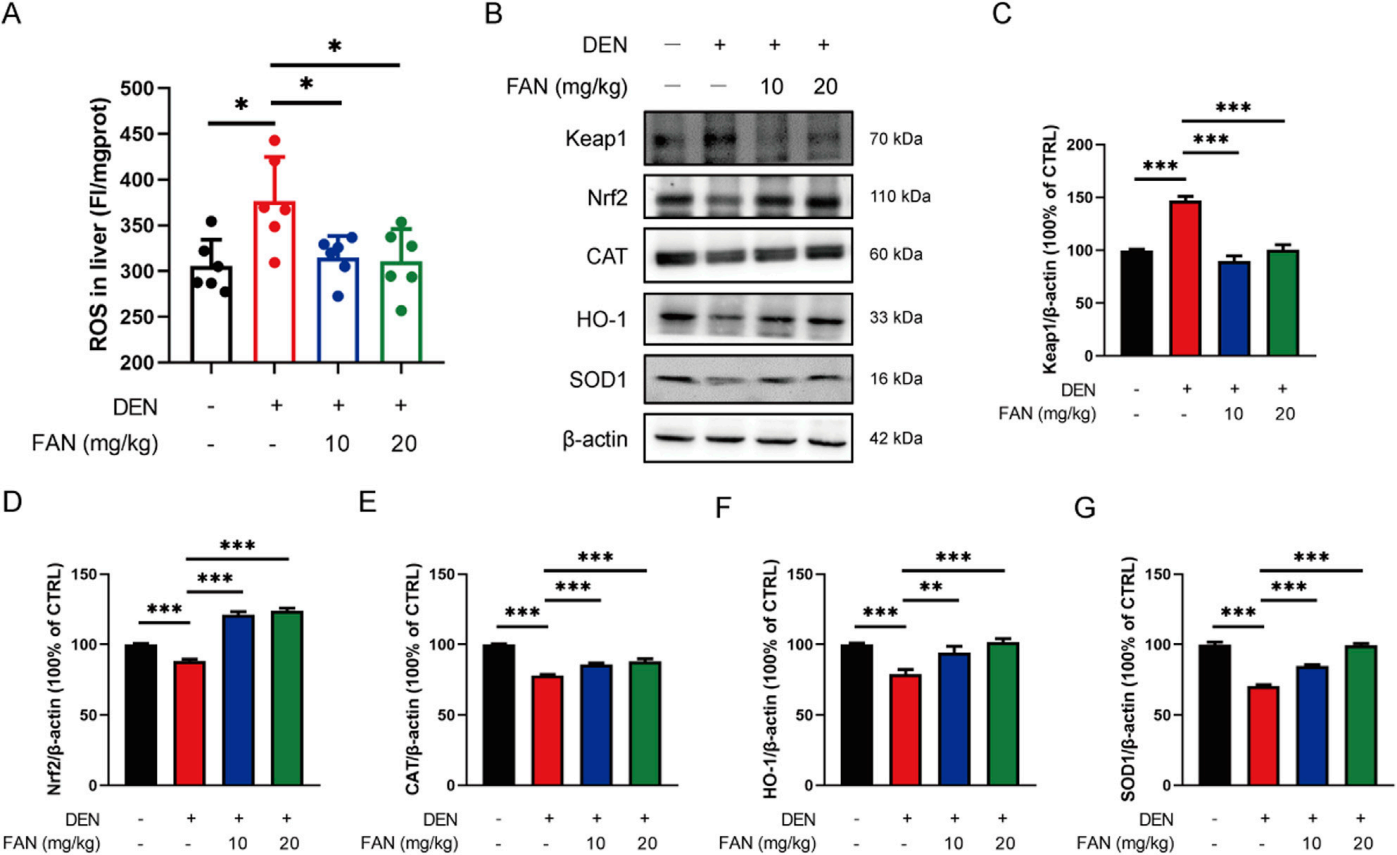
FAN decreases the ROS accumulation by regulating the Nrf2 pathway in mice with liver fibrosis. **(A)** The level of ROS in the liver was detected; n = 6. **(B–G)** Expressions of the indicated proteins in the liver were detected by Western blot **(B)**, and the relative expressions of indicated proteins over β-actin were quantified **(C–G)**; n = 3. Data are presented as the means ± S.D. ^*^ *P* < 0.05, ^**^ *p* < 0.01, and ^***^ *p* < 0.001; ns, not significant.

Studies have shown that taurine can act as an antioxidant. We investigated whether FAN inhibits liver fibrosis by regulating oxidative stress. DEN exposure for 18 weeks resulted in increased ROS levels within the hepatic tissue, which were significantly reduced by FAN treatment (Figure 5A). The Nrf2 signaling pathway is the most important antioxidant mediator. FAN administration alleviated the inhibition of the Nrf2 pathway in the liver specimens of rodents with hepatic fibrosis by increasing the Nrf2 expression and its downstream detoxification enzymes’ (HO-1, SOD1, and CAT) protein levels and decreasing the protein level of Keap1 (Figures 5B–G). This suggests that FAN may inhibit hepatic fibrosis by regulating oxidative stress.

**FIGURE 6:**
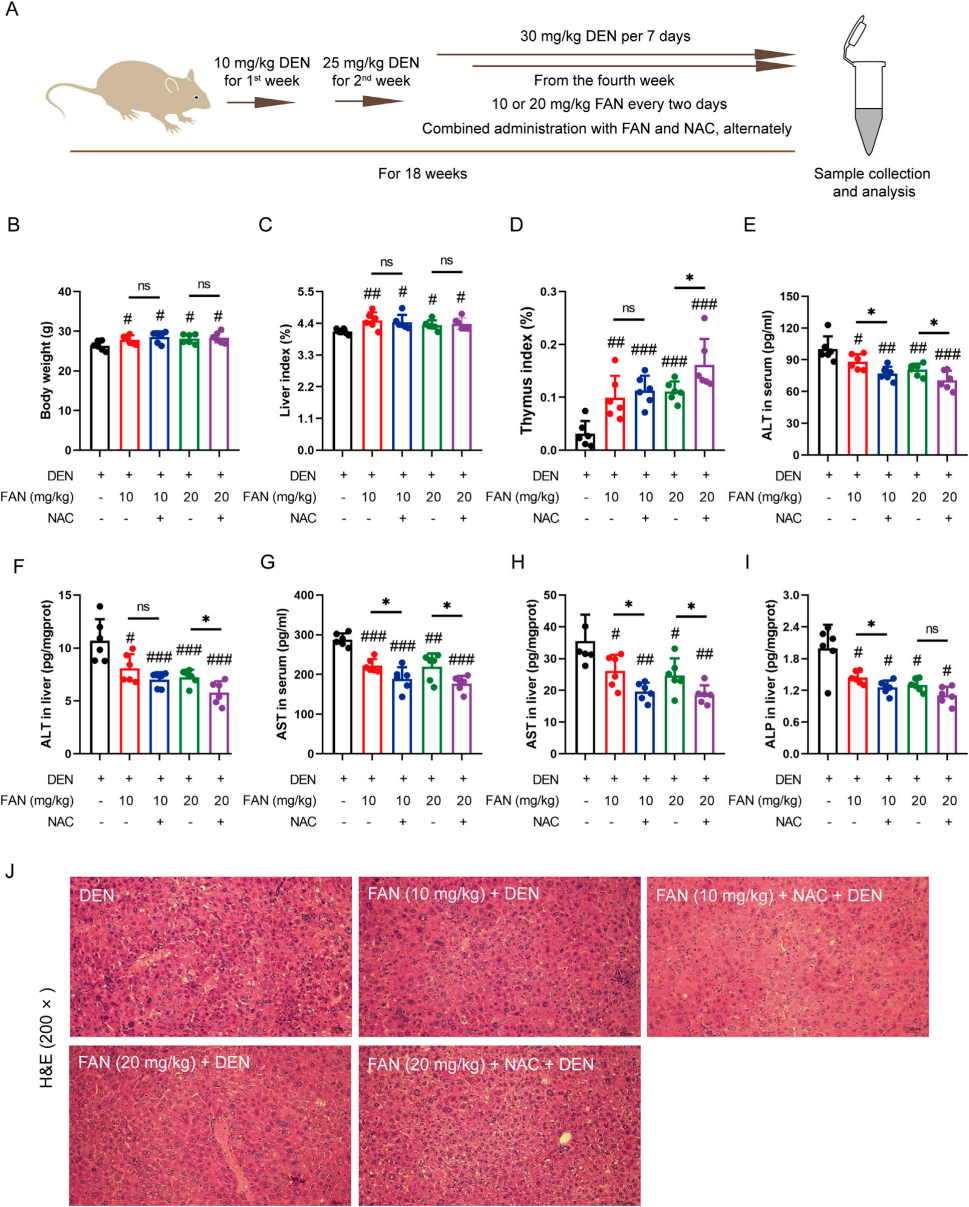
Inhibition of ROS accumulation strengthens the protective effects of FAN against liver injury induced by DEN in the mouse model. **(A)** Diagram of the experimental plan for developing the DEN-induced hepatic fibrosis model and drug treatment (n = 6). **(B)** Body weight was monitored at the end of the experiment. **(C, D)** Indices of the liver and thymus were measured at the time of sacrifice. **(E–I)** Serum or liver ALT, AST, and ALP levels were detected. **(J)** Mouse liver was stained with H&E. Scale bar, 20 μm. Data are presented as the means ± S.D. ^#^ *P* < 0.01, ^##^ *p* < 0.01, and ^###^ *p* < 0.001 in a comparison with the only DEN-treated group; ^*^ *P* < 0.05; ns, not significant.

NAC, a thiol group-containing water-soluble antioxidant, is commonly used to neutralize ROS. We also used DEN to generate a murine model of hepatic fibrogenesis and administered FAN with or without NAC (Figure 6A). We aimed to evaluate the effect of ROS removal on the anti-fibrotic effect of FAN according to the results of liver fibrosis-related indicators *in vivo*. The indices of the heart and thymus were significantly changed after treatment with FAN, and this improvement trend was further reinforced after combination treatment with NAC and FAN; however, the synergistic effects of NAC and FAN did not significantly alter the body weight, liver index, spleen, lung, and renal index (Figures 6B–D; Supplementary Figures S2A–D). Based on the same process, the effect of NAC on FAN-mediated ALT, AST, and ALP levels was examined by ELISA. Compared with that in the FAN-treated group, levels of these three markers were more pronouncedly reduced in the co-treated group (Figures 6E–I).

**FIGURE 7:**
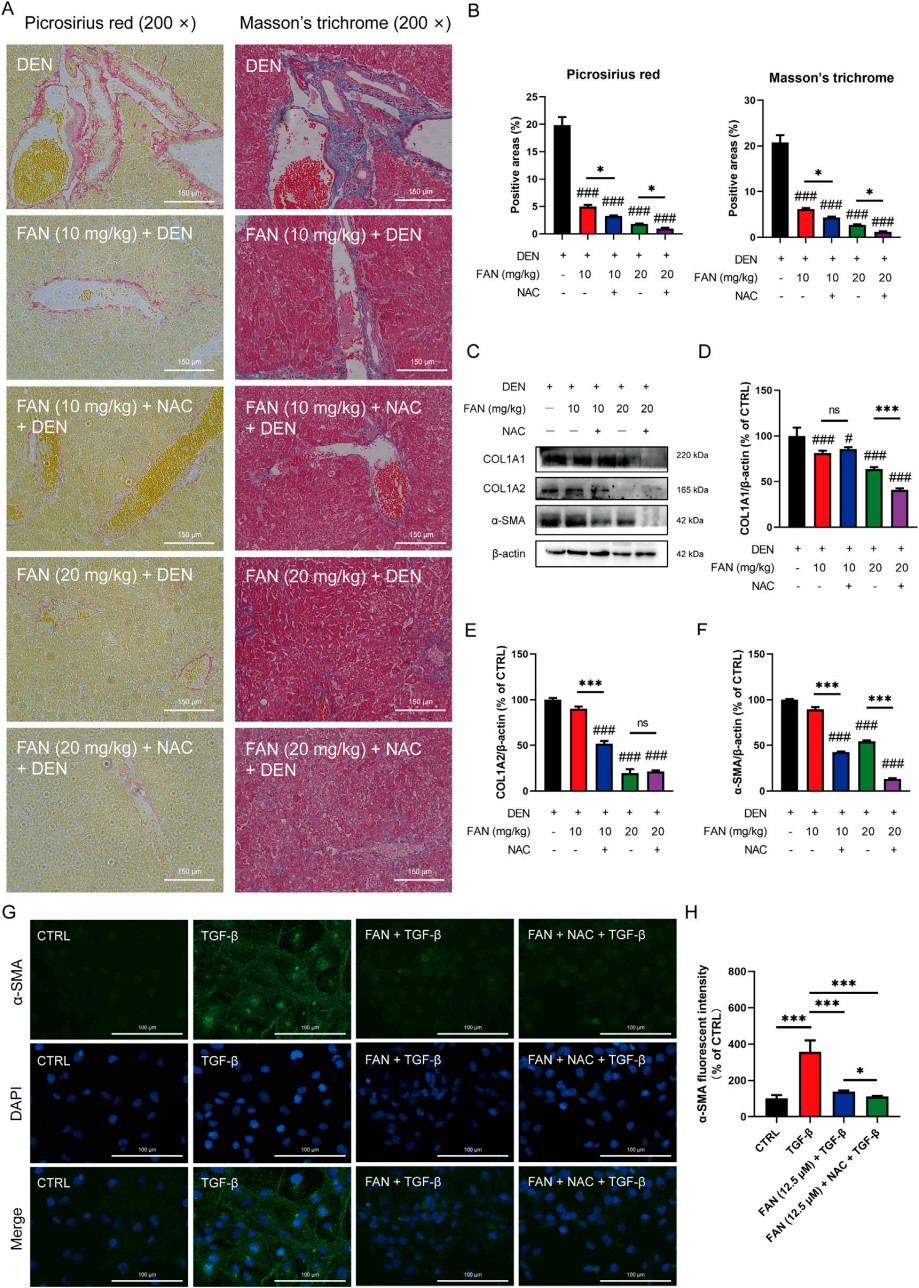
Inhibition of ROS accumulation strengthens the protective effects of FAN against hepatic fibrosis induced by DEN in mice. **(A, B)** Liver tissues of mice were subjected to Picrosirius red staining (A, left panel) or Masson’s trichrome (A, right panel), and quantitative analysis was performed on the positively stained areas **(B)**. Scale bar, 150 μm; n = 3. **(C–F)** Expressions of the indicated proteins in the liver were detected by Western blot **(C)**, and the relative expressions of indicated proteins over β-actin were quantified; n = 3 **(D–F)**. **(G, H)** LX-2 cells were treated with TGF-β for 24 h to establish an activated HSCs model. Then, the cells were treated with 12.5 μM FAN in combination with or without NAC for 24 h. The expression of α-SMA was detected by immunofluorescence **(G)** and quantitatively analyzed **(H)**; scale bar, 100 μm; n = 3 . ^#^ *P* < 0.01 and ^###^ *p* < 0.001 in a comparison with the only DEN-treated group; ^*^ *P* < 0.05 and ^***^ *p* < 0.001; ns, not significant.

The pharmacological suppression caused by FAN on the DEN-induced histopathologic changes in the liver, including hydropic degeneration, swelling, and necrosis of liver cells and inflammatory cell infiltration, was further enhanced under combination treatment (Figure 6J). Combination treatment with NAC and FAN improved DEN-induced deposition of collagen molecules in the liver specimens of rodents better (Figures 7A, B) and further reduced the expression levels of COL1A1, COL1A2, and α-SMA (Figures 7C–F). Consistent with the findings described above, immunofluorescence results demonstrated that co-treatment with NAC further reduced the FAN-mediated decrease in TGF-β-induced α-SMA protein levels in LX-2 cells (Figures 7G, H).

## 4 Discussion

Chronic liver damage may progress through fibrogenesis to end-stage cirrhosis. Hepatic fibrosis manifests as a pathological hallmark of aberrant extracellular matrix remodeling, characterized by excessive collagen deposition along with connective tissue protein dysregulation (Sharma et al., 2023). A key pathomechanism of hepatic fibrogenesis involves the production of oxidative stress in cells by the stimulators (Ruart et al., 2019). FAN, a bibenzyl isoquinoline alkaloid derived from *S. tetrandra S. Moore*, demonstrates multi-pharmacological efficacy, including anti-hepatocellular carcinoma and anti-inflammatory properties (Liu et al., 2024; Zheng et al., 2024). However, whether FAN can alleviate hepatic fibrosis and what its mechanism of action is have not been reported so far. We have clarified the anti-fibrotic effect of FAN based on a murine hepatic fibrosis model and cell model, which was potentially mediated through taurine metabolic modulation and oxidative stress mitigation.

DEN has obvious cytotoxicity and hepatotoxicity (Chi et al., 2016). Its metabolite can cause hypermethylation of nucleic acids and proteins, leading to hepatocyte necrosis. It can also stimulate the synthesis and secretion of the ECM in the liver, thus promoting the formation of fibrosis. The pathological features of DEN-induced liver fibrosis in mice are in line with those of patients with liver fibrosis (Robarts et al., 2023). Here, we confirmed that FAN administration alleviated hydropic degeneration, swelling, and liver cell necrosis and inhibited the deposition of collagen molecules in the liver. DEN-induced hepatotoxicity manifests as dysregulated hepatic enzyme (ALT, AST, and ALP) elevation, with subsequent leakage into systemic circulation, serving as validated indicators of parenchymal injury. FAN effectively reduced serum and liver ALT, AST, and ALP levels, while ameliorating body weight loss and liver index reduction.

Extracellular matrix synthesis and collagen deposition are key events during fibrotic progression (Bataller and Brenner, 2005). HSCs serve as the principal cellular mediators of ECM homeostasis and remodeling in the normal liver (Jiang et al., 2021). They establish intimate contact with surrounding cells (hepatocytes, other stellate cells, and sinusoidal endothelial cells) through cytoplasmic processes. Persistent hepatic injury induces activation of HSCs, driving their transdifferentiation into MF-like cells (HSCs/MFs) (Du et al., 2023). HSCs/MFs exhibit all the characteristics of hepatic MFs, accounting for 82%–96% of all MFs, and trigger the synthesis of abundant ECM components, especially fibrillary collagen. As a cytoskeleton protein, α-SMA is considered to be a marker of myofibroblasts (Wang et al., 2017). We found that FAN not only reduced the collagen deposition and elevation of COL1A1, COL1A2, and α-SMA in fibrotic liver but also decreased the levels of the three proteins in activated HSCs. All these confirmed the anti-fibrotic properties of FAN in hepatic fibrosis.

To clarify the molecular mechanisms underlying FAN’s anti-fibrotic effect, we used proteomic analysis to enrich and analyze abnormally expressed proteins in fibrotic murine hepatic tissues. DEN influenced taurine and hypotaurine metabolism, unsaturated fat synthesis, and arachidonic acid metabolism, among others. This is shown by the fact that the protein level of CSAD, a key enzyme catalyzing taurine synthesis, was significantly decreased in fibrotic liver, as screened by a volcano plot. Notably, FAN administration upregulated CSAD expression and the level of taurine in fibrotic murine hepatic tissue. Taurine, the predominant free amino acid in mammalian systems, demonstrates ubiquitous distribution, with high concentrations observed in the hepatic, renal, muscular, and cerebral compartments (Tramonti et al., 2021). Taurine suppresses HSCs activation through inhibiting autophagy and inducing iron concentration (Li et al., 2024). Activated HSCs undergo phenotypic switching, which causes excessive extracellular matrix deposition and changes in normal liver structure, thereby negatively affecting liver function (Krizhanovsky et al., 2008). Taurine biosynthesis proceeds via a two-step enzymatic cascade: L-cysteine sulfinic acid undergoes CSAD-catalyzed decarboxylation to form hypotaurine, followed by oxidation to yield taurine, with CSAD serving as the key regulatory enzyme within the pathway (Tramonti et al., 2021). We obtained primary murine activated HSCs and established an activated model of HSCs using TGF-β-induced LX-2 cells, while treating cells with either taurine and FAN in combination or not. We found that taurine further enhanced the tendency of FAN to inhibit HSCs activation and decrease the protein levels of COL1A1, COL1A2, COL3A1, and α-SMA in HSCs. These results suggest that FAN exerts anti-fibrotic effects through the modulation of taurine biosynthesis enhancement.

Taurine not only binds bile acid (Solaas et al., 2004) but also acts as an antioxidant (Jong et al., 2021) and membrane stabilizer (Murakami, 2015). Oral taurine administration prevents the progression of hepatic fibrosis through mitigating oxidative damage (Miyazaki et al., 2005). Oxidative stress arises from ROS–antioxidant dysregulation, marked by compromised free radical scavenging capacity and consequent ROS accumulation (Lai et al., 2024). Excessive accumulation of ROS impairs hepatic cellular function and significantly contributes to liver fibrogenesis. ROS can promote HSCs activation (Zhang et al., 2017). ROS can also stimulate hepatocyte necrosis and apoptosis, resulting in liver injury, which aggravates end-stage liver disease (Marañón et al., 2024). Nrf2, a bZIP family transcription factor, activates antioxidant defense mechanisms through interaction with antioxidant response elements in regulatory regions of target genes including SOD and HO-1 (Hayes et al., 2020). SOD catalyzes the dismutation of superoxide radicals into the less reactive hydrogen peroxide radical and inhibits oxidative inactivation of nitric oxide to prevent the formation of peroxynitrite (Tian et al., 2021). HO acts as an indirect antioxidant by blocking the production of free radicals from heme (Dennery, 2014). Normally, Nrf2 proteins bind to Keap1 and are rapidly degraded after ubiquitination (Villeneuve et al., 2013). If Keap1 is oxidized or degraded in response to external stimuli, it releases Nrf2. Following nuclear translocation, Nrf2 orchestrates the initiation of the transcription of antioxidant genes (Gorrini et al., 2013). We observed that FAN treatment suppressed the elevation in the level of ROS induced by DEN exposure; moreover, it was able to increase the expressions of Nrf2 and its downstream associated proteins and decrease the protein level of Keap1, thus preventing the inhibition of the Nrf2 pathway in the progression of liver fibrosis. Combination therapy with FAN and the ROS scavenger NAC demonstrated synergistic anti-fibrotic effects, as evidenced by the significantly attenuated hepatic collagen deposition. This suggests that the anti-fibrotic effects of FAN may depend on Nrf2 pathway activation-mediated oxidative stress attenuation.

## 6 Conclusion

This study demonstrates that FAN inhibits hepatic fibrosis progression in both *in vivo* and *in vitro* models, primarily through modulating taurine biosynthesis and restoring oxidative stress homeostasis. Furthermore, we delineate the molecular pathways underlying FAN’s anti-fibrotic activity, thus providing a pharmacological foundation for novel therapeutic development targeting fibrotic pathogenesis.

## Data Availability

The original contributions presented in the study are included in the article/Supplementary Material, further inquiries can be directed to the corresponding author.
