# Supplementary material for: Fangchinoline attenuates hepatic fibrosis by regulating taurine metabolism and oxidative stress
Source: Front Pharmacol. 2025 Aug 20;16:1633519. doi: 10.3389/fphar.2025.1633519 (PMC12405315; doi:10.3389/fphar.2025.1633519)
Supplement: Supplementary file 1 [file DataSheet1.pdf]

Fangchinoline attenuates hepatic fibrosis by regulating taurine metabolism and oxidative stress

Hui Yin[1, 2], Hang Lian[1], Yawen Wang[1], Luoting Chen[1], Xueting Liu[1], Yange Liu[1,*]

[1]School of Basic Medical Sciences, Jiangxi Medical College, Nanchang University, Nanchang, Jiangxi 330031, China.

[2]Department of Thoracic Surgery, The First Affiliated Hospital of Shaoyang University, Shaoyang, 422000, China.

[*]Corresponding author. Dr. Yange Liu (liuyange@ncu.edu.cn), School of Basic Medical Sciences, Jiangxi Medical College, Nanchang University, Nanchang, 330031 P. R. China.

Figure legend

Figure S1. FAN alleviates DEN-induced the toxicity of organ. (A) Developing DEN-induced HCC mouse model (n=6), after FAN-treatment for 15 weeks, liver was collected and measured at time of sacrificed. (B and C) The lung (B) and kidney (C) were fixed, dehydrated and embedded in paraffin and sliced into 5 μm sections. Thin sections of mouse liver were stained with H&E. Scale bar, 250 μm. Data are means ± S.D..

Figure S2. Inhibition of ROS accumulation partly affects the regulatory effect of FAN on organ index. Organ index was measured at time of sacrificed including cardiac index (A), spleen index (B), lung index (C) and renal index (D). Data are means ± S.D. $^{\#}$ $P < 0.01$, $^{\#\#}$ $P < 0.01$ and $^{\#\#\#}$ $P < 0.001$ in a comparison with the control group; * $P < 0.05$.

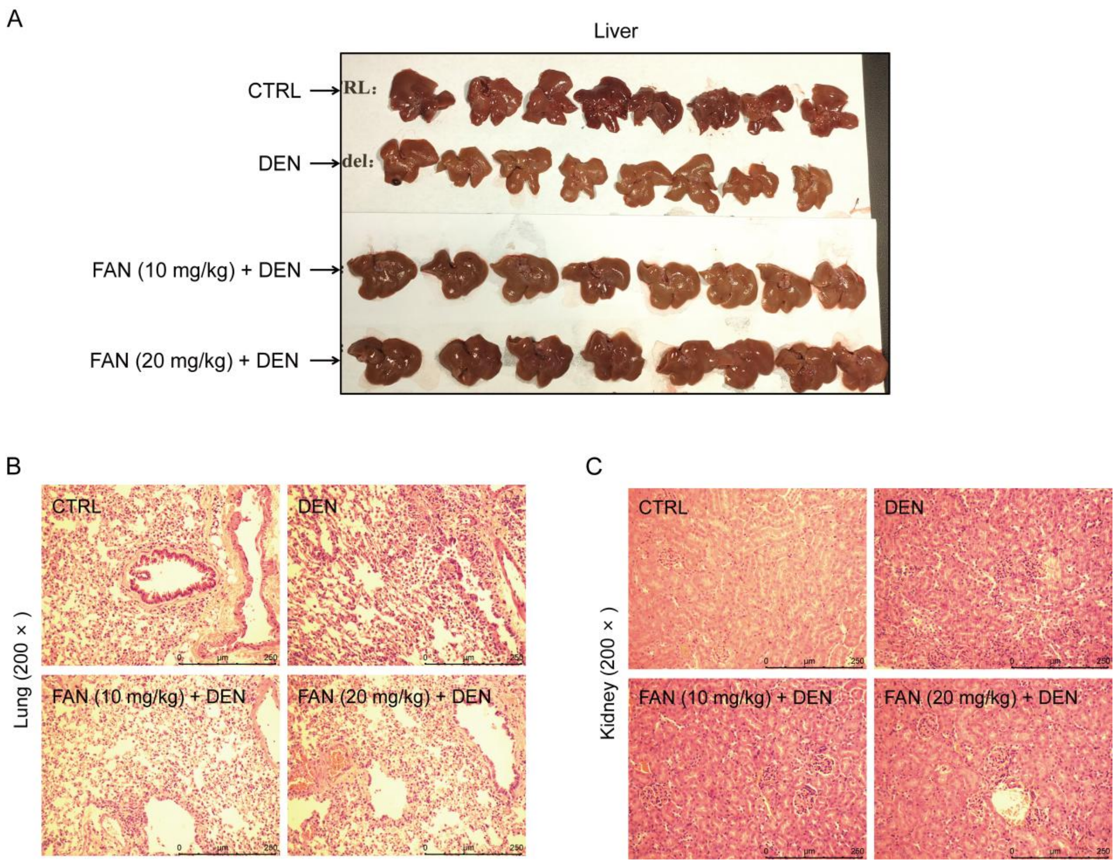

A
Liver
CTRL
RL:
DEN
del:
FAN (10 mg/kg) + DEN
FAN (20 mg/kg) + DEN
B
Lung (200 ×)
CTRL
DEN
FAN (10 mg/kg) + DEN
FAN (20 mg/kg) + DEN
C
Kidney (200 ×)
CTRL
DEN
FAN (10 mg/kg) + DEN
FAN (20 mg/kg) + DEN

Figure S1

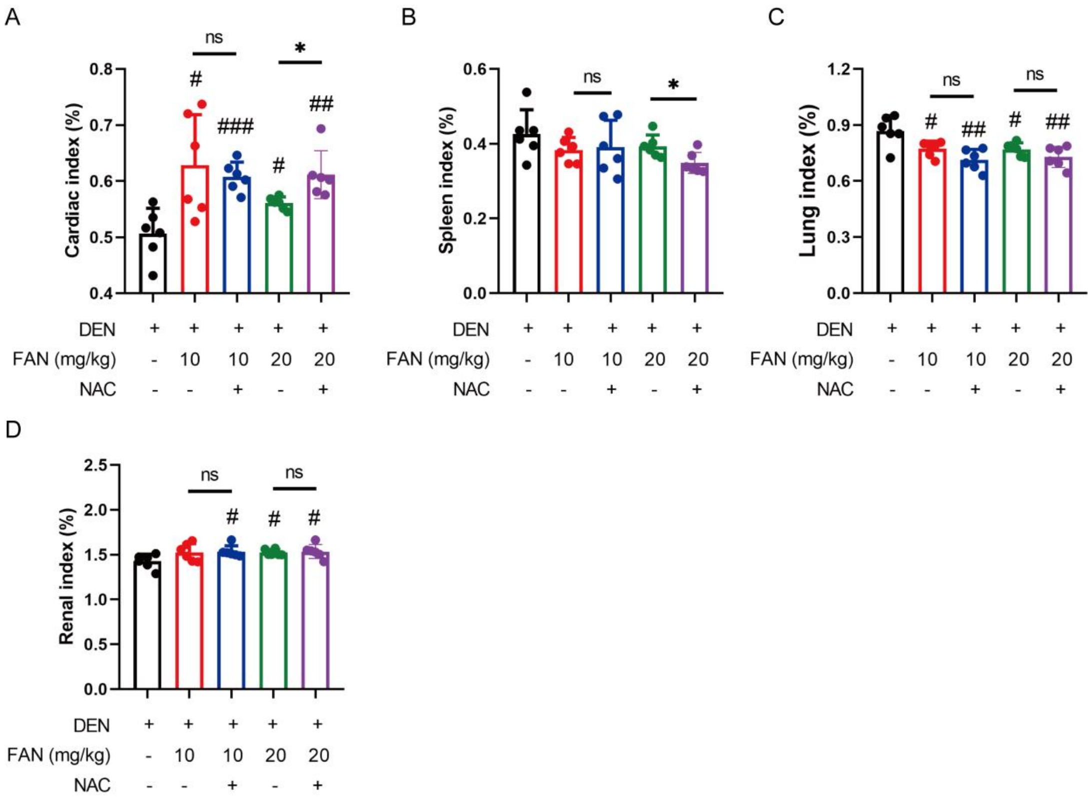
A
Cardiac index (%)
ns
*
#
###
#
##
DEN
FAN (mg/kg)
NAC
B
Spleen index (%)
ns
*
C
Lung index (%)
ns
ns
#
##
#
##
D
Renal index (%)
ns
ns
#
#
#

Figure S2
